# Fertility and Symptom Relief following Robot-Assisted Laparoscopic Myomectomy

**DOI:** 10.1155/2015/967568

**Published:** 2015-04-19

**Authors:** Michael C. Pitter, Serene S. Srouji, Antonio R. Gargiulo, Leslie Kardos, Usha Seshadri-Kreaden, Helen B. Hubert, Glenn A. Weitzman

**Affiliations:** ^1^Department of Obstetrics & Gynecology, Columbia University Medical Center, New York, NY 10032, USA; ^2^Department of Obstetrics and Gynecology, Center for Infertility and Reproductive Surgery, Brigham and Women's Hospital, Harvard Medical School, 75 Francis Street, Boston, MA 02115, USA; ^3^Department of Obstetrics and Gynecology, California Pacific Medical Center, 475 Brannan Street, San Francisco, CA 94107, USA; ^4^Department of Clinical Affairs, Intuitive Surgical Inc., 1266 Kifer Road, Building 101, Sunnyvale, CA 94086, USA; ^5^Stanford University School of Medicine Emerita, 1043 Oakland Avenue, Menlo Park, CA 94025, USA; ^6^Nashville Fertility Center, 345 23rd Avenue, Nashville, TN 37203, USA

## Abstract

*Objective*. To examine success of robot-assisted laparoscopic myomectomy (RALM) measured by sustained symptom relief and fertility. *Methods*. This is a retrospective survey of 426 women who underwent RALM for fibroids, symptom relief, or infertility at three practice sites across the US. We examined rates of symptom recurrence and pregnancy and factors associated with these outcomes. *Results*. Overall, 70% of women reported being symptom-free, with 62.9% free of symptoms after three years. At >3 years, 66.7% of women who underwent surgery to treat infertility and 80% who were also symptom-free reported achieving pregnancy. Factors independently associated with symptom recurrence included greater time after surgery, preoperative dyspareunia, multiple fibroid surgeries, smoking after surgery, and preexisting diabetes. Factors positively correlated with achieving pregnancy included desiring pregnancy, prior pregnancy, greater time since surgery, and Caucasian race. Factors negatively correlated with pregnancy were advanced age and symptom recurrence. *Conclusions*. This paper, the first to examine symptom recurrence after RALM, demonstrates both short- and long-term effectiveness in providing symptom relief. Furthermore, RALM may have the potential to improve the chance of conception, even in a population at high risk of subfertility, with greater benefits among those who remain symptom-free. These findings require prospective validation.

## 1. Introduction

Myomas are benign tumors of the smooth muscle cells of the myometrium. The incidence of myomas increases with age, reaching 40–60% of women by age 35 [1]. Although some myomas are asymptomatic, others can cause bleeding, pain, urinary symptoms, and subfertility, leading women to seek treatment. Myomectomy serves as an effective, fertility-preserving option for the surgical treatment of uterine leiomyomas, although its precise role in enhancing fertility remains controversial [2].

Conventional laparoscopic myomectomy (LM) provides a minimally invasive alternative and results in reduced blood loss, incidence of fever, hospital stay, and convalescence time when compared to abdominal myomectomy (AM); with similar symptom relief and pregnancy outcomes [3, 4] there are surgical challenges associated with LM that have limited patient eligibility and widespread adoption [5].

In 2005, the Food and Drug Administration approved the da Vinci Surgical System for gynecologic indications (Intuitive Surgical, Inc., Sunnyvale, CA, USA). Surgical ergonomics, high definition 3-dimensional vision, and wrist-like flexibility of the instrumentation expanded the range and complexity of cases that could be reliably completed laparoscopically. A recent meta-analysis reported significant short-term benefits with robot-assisted laparoscopic myomectomy (RALM) when compared to AM, including reduced blood loss, fewer blood transfusions and fevers, and a reduced hospital stay [6].

However, long-term outcomes following RALM, in particular symptom recurrence and pregnancy rates, have received little attention thus far. Fertility following RALM has been reported only in a few small case series [7–9]. Pitter et al. [10] studied outcomes of pregnant women following RALM in a large patient series but made no reference to women who did not achieve pregnancy. Thus, no pregnancy rates or correlates of success could be determined. The objective of this large patient study was to determine the long-term success of myomectomy as measured by rates of symptom relief and pregnancy over time, as well as factors associated with the recurrence of symptoms and the likelihood of achieving pregnancy following RALM.

## 2. Methods

This multicenter study included investigators and participants from three gynecology practices in the northeast, southeast, and western US. One practice specialized in treating infertility (Nashville Fertility Center, GW) while the other two larger academic centers (Newark Beth Israel Medical Center, MP; California Pacific Medical Center, KL) provided all gynecologic care.

### 2.1. Ethical Approval

This study was conducted according to the Declaration of Helsinki for Medical Research Involving Human Subjects. The Institutional Review Board at each of the sites approved the study design and execution.

### 2.2. Participants

All women who had previously undergone RALM under the supervision of any one of the investigators were eligible to participate in the study. Eligible patients with current phone numbers were contacted and given an explanation about the study and what participation would entail. Women who consented were then emailed a link to the structured survey or, if preferred, sent a hard copy of the instrument by post, along with unique subject and site identification numbers. Nonrespondents were contacted a maximum of two more times, either electronically or by phone, and were again asked to participate. The study purpose, maintenance of confidentiality, and consent via questionnaire completion were further discussed on an introductory page of the survey.

### 2.3. Data Collection

Patients who had RALM between August 2005 and November 2013 were contacted to participate. Surveys were completed during a 9-month period between September 2013 and May 2014. MarketTools (http://www.markettools.com) administered the online survey, and paper forms were later transcribed into the online database. All information was downloaded from the website and stored in a secure, encrypted database. Only deidentified data were collected and investigators and research staff alone were permitted access.

Information was collected on each patient's primary reason for undergoing myomectomy: to treat symptoms with no intention of becoming pregnant, to treat symptoms with the possibility of becoming pregnant in the future, or to improve the current chance of pregnancy. Additional variables of interest included patient information and sociodemographic characteristics at the time of survey completion (months since the myomectomy, age, height, weight, and ethnicity), status prior to myomectomy (smoking status, preexisting medical conditions, pregnancies and their outcomes, attempted pregnancy without success, infertility treatments, symptoms or conditions that caused patients to seek treatment, and prior surgery for fibroids), and postsurgical information (smoking status, time waited before attempting pregnancy, achieved pregnancy and outcomes, time until achieving pregnancy, use of fertility interventions, complications of pregnancy, and symptom or condition recurrence).

### 2.4. Statistical Analysis

Analyses were performed on completed surveys only, although skipped questions were permissible. Data were stratified into three groups defined by each participant's primary reason for undergoing myomectomy: group 1: to treat symptoms with no intention of becoming pregnant, group 2: to treat symptoms with the possibility of becoming pregnant in the future, and group 3: to improve the current chance of pregnancy. Using this stratification, variables were described by means and standard deviations or percentages, as appropriate. Analyses, further stratified by respondents' time since myomectomy, included rates of symptom remission (groups 1, 2, and 3) and pregnancy (groups 2 and 3 only). Tests for trends in the percentage of women who were symptom-free over time and the percentage of women who achieved pregnancy over time were conducted using a Cochrane-Armitage trend test. Multivariable forward stepwise logistic regression was used to identify patient sociodemographic factors, preexisting conditions, and other pre- and postmyomectomy characteristics significantly associated (two-sided *P* < 0.05) with symptom recurrence and, for groups 2 and 3, achieving pregnancy. Results are described using the odds ratio (OR) and 95% confidence interval (95% CI). All analyses were done using SAS version 9.2.1 (SAS Inc., Cary NC).

## 3. Results

### 3.1. Survey Response Rate

Invitations to participate were extended to 852 women who had undergone RALM and had current phone numbers; 59.0% requested to complete the survey online and 41.0% preferred paper forms that were sent and returned by post. The survey response rate was 50%; thus, data were available for analysis from 426 patients with a prior RALM. The average time between myomectomy and survey completion was approximately 2.5 years and ranged from 1 month to 7.5 years.

### 3.2. Patient Characteristics

Women in group 1, who only wanted symptom treatment and had no intention of becoming pregnant, comprised 19.2% of the cohort. The largest group was group 2 (66.0%) and included those who wanted symptom treatment with the possibility of becoming pregnant in the future. Group 3, women desiring to improve their current chances of pregnancy, was the smallest (14.8%). At the time of the survey, the average participants' age was 40.4 ± 6.1 years, most women being self-identified as African American (46.5%) or as Caucasian (38.2%). BMI was 27.0 ± 6.0 kg/m^2^ and the majority of women (92.9%) were nonsmokers. Among those who possibly or definitely desired pregnancy (groups 2 and 3, resp.), infertility after myomectomy was diagnosed in about 8% overall and 16% in group 3 alone.

Age at the time of myomectomy was 37.9 ± 5.8 years (Table 1). Smoking was prevalent in 16.3%. Preexisting conditions included anemia (20.9%), endometriosis (13.3%), hypertension (9.0%), and diabetes (2.4%). The two most common reasons for seeking treatment included excessive menstrual bleeding (51.6%) and pain/pressure (48.8%). However, 21% did not report either of these symptoms or infertility as reasons for RALM. About 15% of women had undergone two or more prior surgeries for fibroid treatment.

While 53% of women had become pregnant prior to their robotic myomectomy, 43% had attempted but not conceived during this time (Table 1). More than one-third of the women who had prior pregnancies experienced miscarriages (36%), with the highest rates in groups 2 and 3. Infertility treatments were obtained by 15% of women overall and 27% in group 3 alone.

### 3.3. Symptom Relief

Approximately 70% of participants reported that they were symptom-free at the time of survey completion. The overall proportion of women who were symptom-free decreased as the interval of time since surgery increased: 81.3% were symptom-free up to 12 months, 72% at 12–24 months, 64.8% at 24–36 months, and 62.9% at greater than 3 years from surgery. Symptom recurrences were mostly excessive menstruation (52%) and pain/pressure (48%). One patient (0.3%) reported having received additional treatment after her original RALM. This group 2 patient underwent an AM two years after her robotic myomectomy.

**Figure 1:**
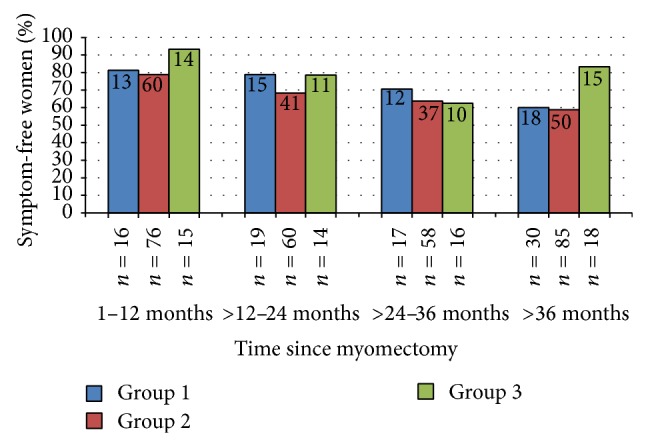
Women who were symptom- and condition-free since myomectomy, by group and time since myomectomy. Group 1 (blue bars) desired to treat symptoms with no intention of becoming pregnant; group 2 (red bars) desired to treat symptoms and preserve ability to get pregnant in the future; group 3 (green bars) desired to improve current chance of pregnancy after myomectomy. *N* in bars is the number of symptom-free women; *N* below the bars is the total number of women in that group and time period.

The group 1 patients who sought treatment solely for the purpose of symptom relief best demonstrate the effectiveness of robotic surgery to improve fibroid-related symptoms. The proportion of women who were symptom-free in this group also decreased as the time from surgery increased (Figure 1): 81.3% were symptom-free at 1–12 months, 78.9% at 12–24 months, 70.6% at 24–36 months, and 60% at greater than 3 years (trend test *P* = 0.041).

Women in group 3, who had surgery to improve fertility, had the greatest relief of symptoms and the lowest rate of symptom recurrence over time. While the trend in symptom relief over time for group 2 women who had surgery for symptom relief and fertility preservation was statistically significant (*P* = 0.003), the difference in trends between groups 1 and 2 was not statistically significant (*P* = 0.483), nor were there differences in trends between group 3 and group 2 (*P* = 0.576) or between group 3 and group 1 (*P* = 0.161).

**Figure 2:**
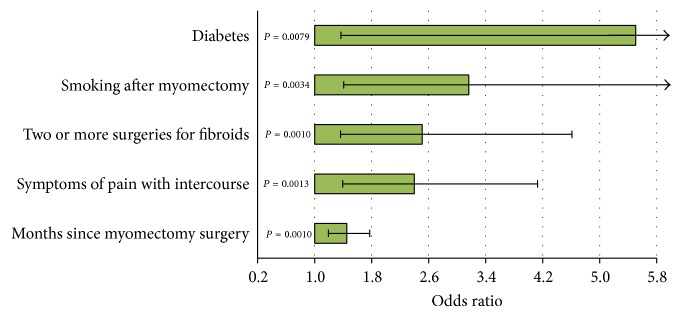
Factors independently associated with recurrence of symptoms. Shown are the factors that were statistically significant at *P* < 0.05 in forward stepwise logistic regression.

Characteristics that were independently associated with symptom recurrence were identified using stepwise logistic regression (Figure 2).

Variables examined included group status, age at myomectomy, months between surgery and survey, ethnicity, BMI, smoking after myomectomy, preexisting medical conditions, symptoms, and multiple fibroid surgeries. Factors associated with recurrence were longer time between myomectomy and survey (OR = 1.45; 95% CI = 1.19, 1.77), prior symptoms of pain with intercourse (OR = 2.40; 1.40, 4.13), two or more fibroid surgeries (OR = 2.51; 95% CI = 1.37, 4.61), smoking after myomectomy (OR = 3.16; 1.41, 7.11), and preexisting diabetes (OR = 5.50; 1.37, 22.11). Although only 2.4% of women had diabetes at myomectomy, diabetics were over five times more likely to have a recurrence of symptoms. The probability of recurrence increased 45% with each 12-month period after surgery.

### 3.4. Achieving Pregnancy

**Figure 3:**
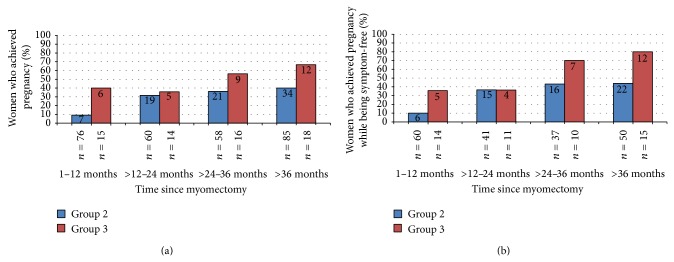
Pregnancy status after myomectomy in women who desired pregnancy, by group and time between myomectomy and survey. Group 2 (blue bars) desired to treat symptoms and preserve ability to get pregnant in the future; group 3 (red bars) desired to improve current chance of pregnancy after myomectomy. *N* in bars is the number of women who became pregnant; *N* below the bars is the total number of women in that group and time period. (a) All women and (b) women who were symptom-free.

In order to assess the impact of surgery on fertility potential, it was necessary to limit the analysis to groups 2 and 3, those who desired pregnancy in the future or currently. These groups differed somewhat in their* a priori* risk of subfertility. Patients in group 3 were older at surgery (mean 38.0 ± 4.9 years versus 36.5 ± 5.5 years, *P* = 0.029) and had a significantly higher rate of infertility as a reason for treatment (60.3% versus 14.2%, *P* < 0.0001), prior miscarriage (58.6% versus 40.6%, *P* = 0.013), and use of preoperative infertility treatments (26.5% versus 12.3%, *P* = 0.006). Despite these poor prognostic indicators, more than half (50.8%) of group 3 patients achieved pregnancy. The pregnancy rates increased with follow-up time after surgery in both groups (Figure 3(a)) and were even higher when restricted to women who were symptom-free (Figure 3(b)).

For the women who achieved pregnancy, the mean time to pregnancy after starting to attempt was 7.9 ± 9.4 months (Table 2). Women waited an average of 4.3 ± 3.1 months before trying to conceive and, thus, time between myomectomy and pregnancy was 12.3 ± 10.3 months. A larger percentage of women in group 3 used medications to achieve pregnancy compared to group 2 (46.9% versus 20%, *P* = 0.007). Although women in group 3 were two years older than women in group 2, major complications during pregnancy (abnormal placentation, uterine rupture, or premature delivery) were lower in group 3 (3.2% versus 15.6%, *P* = 0.103). Those with complications were two years younger on average than the group as a whole. The proportions that miscarried during pregnancy in groups 2 and 3 were 30.5% and 37.5%, respectively.

**Figure 4:**
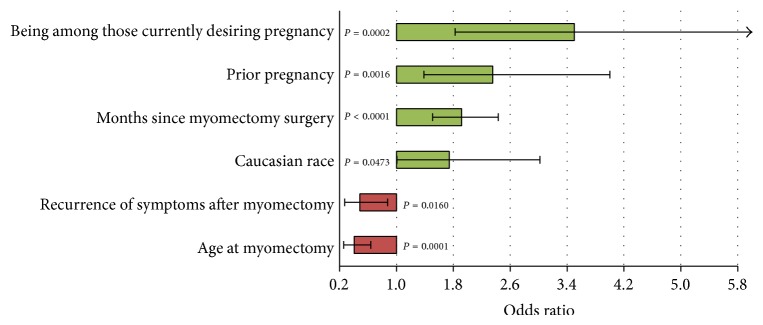
Factors independently associated with achieving pregnancy. Shown are the factors that were statistically significant at *P* < 0.05 in forward stepwise logistic regression.

Logistic regression was used to examine characteristics independently associated with achieving pregnancy after myomectomy in groups 2 and 3 (Figure 4).

Variables chosen included group status, age at myomectomy, months between surgery and survey, ethnicity, BMI, smoking after myomectomy, preexisting conditions, infertility as a reason for myomectomy, pregnancy prior to myomectomy, symptom recurrence after myomectomy, and multiple fibroid surgeries. Characteristics that were significantly and independently associated with achieving pregnancy included Caucasian race (OR = 1.74; 95% CI = 1.01, 3.02), longer time between myomectomy and survey (OR = 1.92; 95% CI = 1.51, 2.43), prior pregnancy (OR = 2.35; 95% CI = 1.39, 4.00), and group 3 status (actively desiring pregnancy) (OR = 3.50, 95% CI = 1.83, 6.71). Older age at myomectomy (OR = 0.41; 95% CI = 0.26, 0.64) and symptom recurrence (OR = 0.49; 95% CI = 0.27, 0.87) were negatively associated with achieving pregnancy. Each increasing decade of age at myomectomy (20–29, 30–39, 40–49, etc.) decreased the odds of pregnancy by 41%.

## 4. Discussion

### 4.1. Principal Findings

We have shown long-term treatment success for the majority of women undergoing RALM. Relief from symptoms was achieved in 70% of all patients, with 62.9% of women still symptom-free more than three years after surgery. Pregnancy rates were high in those actively trying to conceive (50.8% overall and 66.7% after more than three years postoperatively), despite this group's age and subfertility. Moreover, staying symptom-free increased the chances of conception, with an 80% pregnancy rate for symptom-free patients in group 3 after more than three-year follow-up. These results suggest a sustained benefit of RALM on fertility potential and patient comfort.

### 4.2. Relation to Other Studies

This multicenter study is among the first to examine the long-term success of RALM. There have been only a few papers examining pregnancy rates following RALM, and no published studies of symptom recurrence were identified in our searches. Cela and colleagues [7] reported an overall pregnancy rate of 13% in 48 patients, with a success rate of 77.8% in 9 women desiring pregnancy. In the study by Lonnerfors and Persson [8], 15 out of 22 (68.2%) women who actively wanted to achieve pregnancy were successful after removal of deep intramural myomas by RALM. Tusheva et al. [9] reported a success rate of 75% in 16 RALM patients desiring pregnancy. We have previously reported on pregnancies following RALM in a large, multicenter study [10] and found an overall conception rate of 12.3%, similar to the rate of 13% shown by Cela et al. [7]. However, it is unknown how many women were actively trying to conceive.

In the present study, the pregnancy rate was 26.8% for all women, including those not desiring pregnancy after surgery. This rate is twice that seen in Cela et al.'s study [7] and in our own prior study [10]. The difference may reflect the larger proportion of women in this study who were actively trying to conceive. Our conception rate after three years among women actively trying to conceive (67%) is on par with the rates from studies discussed above, 68%–78% [7–9]. Average time to pregnancy in our study was 10.6 months, comparable to the median time of 10 months observed by Lonnerfors and Persson [8]. Self-reported miscarriage rates in this study (32%) were higher than those reported in the other smaller RALM studies (0–17%) described above.

RALM may result in improved conception rates relative to conventional LM and AM. A weighted average conception rate for women actively desiring pregnancy based on our and the abovementioned published RALM studies is 46/65 or approximately 71%. This rate is at the high end of the range of pregnancy rates (33%–75%) reported in a recent review of LM among patients undergoing the procedure for reasons of infertility [3]. In addition, a recent abstract by Celestine and colleagues [11] showed a higher rate of spontaneous conception (58%) after RALM compared to AM (32%). Prospective comparative studies are needed to examine whether RALM may result in improved pregnancy rates among women actively trying to conceive.

Since we were unable to find published reports of symptom recurrence after RALM, we examined the LM literature. Our definition of recurrence was based on the self-reported reappearance of fibroid-related symptoms. Recurrence during the first postoperative year was 19.7%, 28% the second year, 35.2% the third year, and 37.1% after three years. Radosa et al. [12] reported an overall rate of symptom recurrence at 35.7%, on par with the overall rate of 30% in the present study. Two studies of LM with a stricter definition of recurrence requiring the return of symptoms followed by ultrasound confirmation of fibroids found lower recurrence rates at 24 months (4.9% and 12.7%) and 60 months (16.7% and 21.4%) [12, 13]. LM studies that utilized a definition of recurrence requiring only the presence of fibroids on follow-up sonograms reported intermediary rates of recurrence at 24 months (20% and 20%) and 60 months (51.5% and 52.9%) [14, 15]. We would argue that basing the recurrence rate on appearance of symptoms might be more clinically relevant. All of these studies reported an increase in recurrence rates over time, as in our study, and an increased risk of recurrence with the presence of multiple myomas. In the present study there was an increased risk of recurrence in patients with two or more prior fibroid surgeries. While our findings are in line with what has been previously reported following LM, studies are needed to determine if RALM provides any additional benefit.

We found that, in addition to longer time since myomectomy and prior fibroid surgery, symptom recurrence was higher among patients who complained of painful intercourse preoperatively, smoked, and had diabetes. Painful intercourse may correlate with larger myoma size or greater number of myomas, which have been shown to correlate with recurrence [12–15]. To the best of our knowledge, this study is the first to examine the relationships of smoking and diabetes with symptom recurrence following myomectomy. Our findings contradict studies reporting a decreased risk of fibroids with smoking [16–19] thought to be mediated by inducing enzymes that promote estrogen metabolism, thus lowering estrogen levels and impeding myoma development [16]. It is possible that smoking correlated with another, unmeasured factor or that those in pain due to symptom recurrence were more likely to smoke. Findings in one review agree with our results, reporting an increased risk of myomas with diabetes, stating that this is most likely mediated by insulin resistance, increased IGF-1 activity (a growth factor for fibroid cells in vitro), and elevated androgen levels [17]. Other studies have reported a deceased risk of myomas with diabetes [19–21] and suggest that the protective effect comes from the medications taken. For example, insulin and pioglitazone have been shown to have myoma inhibitory effects [20, 22] and IGF-1 levels decrease in treated diabetics [23]. Differences among studies may be due to the complex etiology of diabetes, levels of blood sugar control, and the wide range of diabetes medications and their effects.

We found that, in addition to longer time since surgery and an active desire to conceive (group 3 women), achieving pregnancy was associated with younger age, prior conception success, Caucasian race, and remaining symptom-free. Other RALM [8, 9] and LM [12, 15, 24, 25] studies have reported a negative association between pregnancy and increasing age and a positive association between prior pregnancy and future conception success [1]. The positive effect of Caucasian race may be related to a lower risk of myoma regrowth without symptom recurrence (controlled for in logistic analysis). Epidemiological studies have consistently reported that African American women are at higher risk for myoma development [16, 19]. It is also likely that Caucasian women had greater access to fertility treatments and assisted technologies, improving their chances of conception. Unfortunately, this factor could not be considered in analyses since it was not assessed in all women. The significant negative association between recurrence and achieving pregnancy has been previously shown in two LM studies in which recurrence was defined by reappearance of fibroids on sonography [13, 15]. It is notable that we found a higher pregnancy rate in symptom-free patients at each time interval postoperatively (compared to rates overall and in those who experienced recurrence), with the highest rate (80%) seen after three years in patients actively attempting to conceive (group 3).

### 4.3. Strengths and Weaknesses

To our knowledge, this is the first published study on long-term recurrence rates following RALM and one of the largest RALM studies to report on pregnancy success. Caveats regarding the results pertain to the retrospective, cross-sectional nature of the design. Survey responses for each participant were captured at differing time periods following myomectomy. This may have resulted in the introduction of bias if, for example, women who had more recent robotic myomectomies had better recall. However, such limitations would have affected all study groups similarly. Furthermore, the parameter, “time since myomectomy,” was adjusted for in all logistic analyses. While our results may not be generalizable to all myomectomy patients, the study included a relatively large number of women from three different US locations and two types of practices and, thus, attempted to introduce greater patient heterogeneity and the potential for generalizability. Although it is possible that data from multiple practices may have introduced unmeasured differences influencing the study findings, there was no impact of “practice site” on symptom recurrence or pregnancy success in the logistic analyses. The impact of fertility interventions, perioperative outcomes, and location, number, or size of myomas could not be assessed since this information was not uniformly collected at all study sites.

## 5. Conclusions

We found that 50.8% of women desiring pregnancy were able to achieve it, in spite of the fact that 60.3% reported infertility as the reason for undergoing myomectomy and many were also at high risk for reproductive failure due to advanced reproductive age and other independent risk factors. Symptom relief in that group overall was 79.4%. These findings are encouraging for women with symptomatic fibroid uteri who desire a minimally invasive approach to treatment of their disease while maintaining their comfort and fertility potential. More research, particularly prospective study, is needed to confirm or refute these results.

## Figures and Tables

**Table 1:** Patient characteristics at myomectomy.

Characteristics	Group 1^a^	Group 2^b^	Group 3^c^	Total
Number of patients	*N* = 82	*N* = 281	*N* = 63	*N* = 426
Age at myomectomy, yrs	42.5 ± 5.0^d^	36.5 ± 5.5	38.0 ± 4.9	37.9 ± 5.8
Smoking prior to surgery, *n* (%)	16 (19.8)	40 (14.3)	13 (20.6)	69 (16.3)
Preexisting conditions, *n* (%)^e^	36 (45.0)	131 (47.1)	24 (38.1)	191 (45.4)
Anemia	15 (18.8)	65 (23.4)	8 (12.7)	88 (20.9)
Endometriosis	10 (12.5)	38 (13.7)	8 (12.7)	56 (13.3)
Hypertension	13 (16.3)	22 (7.9)	3 (4.8)	38 (9.0)
Diabetes	1 (1.3)	8 (2.9)	1 (1.6)	10 (2.4)
Heart disease	0	0	0	0
Other	6 (7.5)	13 (4.7)	4 (6.3)	23 (5.5)
Reasons for seeking treatment, *n* (%)^e^				
Fibroids	68 (82.9)	249 (88.6)	52 (82.5)	369 (86.6)
Excessive menstrual bleeding	49 (59.8)	148 (52.7)	23 (36.5)	220 (51.6)
Pain/pressure	40 (48.8)	152 (54.1)	16 (25.4)	208 (48.8)
Frequent urination	22 (26.8)	62 (22.1)	3 (4.8)	87 (20.4)
Infertility	2 (2.4)	40 (14.2)	38 (60.3)	80 (18.8)
Painful intercourse	18 (22.0)	57 (20.3)	5 (7.9)	80 (18.8)
Endometriosis	8 (9.8)	35 (12.5)	7 (11.1)	50 (11.7)
Ovarian cyst	6 (7.3)	21 (7.5)	4 (6.3)	31 (7.3)
Other	1 (1.2)	1 (.4)	0	2 (.5)
Symptoms with fibroids, *n* (%)^e^				
Excessive menstrual bleeding	39 (57.4)	131 (52.6)	22 (42.3)	192 (52.0)
Pain/pressure	32 (47.1)	130 (52.2)	15 (28.9)	177 (48.0)
Infertility	1 (1.5)	36 (14.5)	31 (59.6)	68 (18.4)
Bleeding and pain	24 (35.3)	84 (33.7)	11 (21.2)	119 (32.2)
Bleeding, pain, and infertility	0	14 (5.6)	7 (14.1)	21 (5.7)
None of these	11 (16.2)	56 (22.5)	12 (23.1)	79 (21.4)
≥2 fibroid surgeries, *n* (%)	11 (13.4)	42 (14.9)	9 (14.3)	62 (14.6)
Prior pregnancy, *n* (%)	56 (68.3)	143 (50.9)	29 (46.0)	228 (53.5)
Attempted but did not achieve pregnancy, *n* (%)	12/58 (20.7)	73/163 (44.8)	24/34 (70.6)	109/255 (42.8)
Outcomes of prior pregnancies, *n* (%)^e^				
Vaginal delivery ≥ 1	29 (51.8)	42 (29.4)	4 (13.8)	75 (32.9)
C-section ≥ 1	14 (25.0)	41 (28.7)	10 (34.5)	65 (28.5)
Miscarriage ≥ 1	8 (14.3)	58 (40.6)	17 (58.6)	83 (36.4)
Ectopic ≥ 1	1 (1.8)	4 (2.8)	2 (6.9)	7 (3.0)
Prior infertility treatments, *n* (%)	8/56 (14.3)	20/162 (12.3)	9/34 (26.5)	37/252 (14.7)

^a^Group 1 = desire to treat symptoms with no intention of becoming pregnant.

^b^Group 2 = desire to treat symptoms and preserve ability to get pregnant in the future.

^c^Group 3 = desire to improve current chance of pregnancy after myomectomy.

^d^Data are mean ± standard deviation unless stated otherwise.

^e^Data not mutually exclusive.

**Table 2:** Characteristics of women who achieved pregnancy after myomectomy.

Characteristics	Group 2^a^	Group 3^b^	Total
Months to achieve pregnancy after starting to attempt			
Number of patients	*N* = 77	*N* = 31	*N* = 108
Mean ± SD	8.2 ± 10.1	7.3 ± 7.5	7.9 ± 9.4
Range	(1–60)	(1–32)	(1–60)
Months to achieve pregnancy after myomectomy			
Number of patients	*N* = 82	*N* = 32	*N* = 114
Mean ± SD	13.0 ± 11.1	10.6 ± 8.3	12.3 ± 10.3
Range	(2.5–64.5)	(2.5–33.5)	(2.5–64.5)
Used medications or procedures to achieve pregnancy, *n* (%)	16/80 (20.0)	15/32 (46.9)	31/112 (27.7)
Age at pregnancy, yrs	35.7 ± 5.0^c^	37.7 ± 5.3	36.3 ± 5.1
Age at pregnancy for women with a complication, yrs	34.5 ± 6.2	33.5 ± 0.0	34.4 ± 5.9
Complications during pregnancy, *n* (%)^d^	12 (15.6)	1 (3.2)	13 (12.0)
Premature delivery <37 wks	10 (13.0)	1 (3.2)	11 (10.2)
Abnormal placentation	4 (5.2)	0	4 (3.7)
Uterine rupture	1 (1.3)	0	1 (0.9)
Miscarriage during pregnancy, *n* (%)	25/82 (30.5)	12/32 (37.5)	37/114 (32.5)

^a^Group 2 = desire to treat symptoms and preserve ability to get pregnant in future.

^b^Group 3 = desire to improve current chance of pregnancy after myomectomy.

^c^Data are mean ± standard deviation unless stated otherwise.

^d^Data are not mutually exclusive.

## References

[1] Parker W. H. (2007). Etiology, symptomatology, and diagnosis of uterine myomas. *Fertility and Sterility*.

[2] Brady P. C., Stanic A. K., Styer A. K. (2013). Uterine fibroids and subfertility: an update on the role of myomectomy. *Current Opinion in Obstetrics and Gynecology*.

[3] Buckley V., Nesbitt-Hawes E. M., Atkinson P. (2015). Laparoscopic myomectomy: clinical outcomes and comparative evidence. *Journal of Minimally Invasive Gynecology*.

[4] Jin C., Hu Y., Chen X. C. (2009). Laparoscopic versus open myomectomy—a meta-analysis of randomized controlled trials. *European Journal of Obstetrics & Gynecology and Reproductive Biology*.

[5] Walid S. M., Heaton R. L. (2011). The role of laparoscopic myomectomy in the management of uterine fibroids. *Current Opinion in Obstetrics and Gynecology*.

[6] Pundir J., Pundir V., Walavalkar R., Omanwa K., Lancaster G., Kayani S. (2013). Robotic-assisted laparoscopic vs abdominal and laparoscopic myomectomy: systematic review and meta-analysis. *Journal of Minimally Invasive Gynecology*.

[7] Cela V., Freschi L., Simi G. (2013). Fertility and endocrine outcome after robot-assisted laparoscopic myomectomy (RALM). *Gynecological Endocrinology*.

[8] Lonnerfors C., Persson J. (2011). Pregnancy following robot-assisted laparoscopic myomectomy in women with deep intramural myomas. *Acta Obstetricia et Gynecologica Scandinavica*.

[9] Tusheva O. A., Gyang A., Patel S. D. (2013). Reproductive outcomes following robotic-assisted laparoscopic myomectomy (RALM). *Journal of Robotic Surgery*.

[10] Pitter M. C., Gargiulo A. R., Bonaventura L. M., Lehman J. S., Srouji S. S. (2013). Pregnancy outcomes following robot-assisted myomectomy. *Human Reproduction*.

[11] Celestine C., Ziegler W., Johnson V., Kuo Y.-H., Mann J. (2014). Pregnancy after abdominal versus robotically assisted laparoscopic myomectomy. *Fertility and Sterility*.

[12] Radosa M. P., Owsianowski Z., Mothes A. (2014). Long-term risk of fibroid recurrence after laparoscopic myomectomy. *European Journal of Obstetrics & Gynecology and Reproductive Biology*.

[13] Doridot V., Dubuisson J.-B., Chapron C., Fauconnier A., Babaki-Fard K. (2001). Recurrence of leiomyomata after laparoscopic myomectomy. *Journal of the American Association of Gynecologic Laparoscopists*.

[14] Nezhat F. R., Roemisch M., Nezhat C. H., Seidman D. S., Nezhat C. R. (1998). Recurrence rate after laparoscopic myomectomy. *The Journal of the American Association of Gynecologic Laparoscopists*.

[15] Yoo E.-H., Lee P. I., Huh C.-Y., Kim D.-H., Lee B.-S., Lee J.-K. (2007). Predictors of leiomyoma recurrence after laparoscopic myomectomy. *Journal of Minimally Invasive Gynecology*.

[16] Faerstein E., Szklo M., Rosenshein N. (2001). Risk factors for uterine leiomyoma: a practice-based case-control study. I. African-American heritage, reproductive history, body size, and smoking. *American Journal of Epidemiology*.

[17] Okolo S. (2008). Incidence, aetiology and epidemiology of uterine fibroids. *Best Practice & Research: Clinical Obstetrics & Gynaecology*.

[18] Ross R. K., Pike M. C., Vessey M. P., Bull D., Yeates D., Casagrande J. T. (1986). Risk factors for uterine fibroids: reduced risk associated with oral contraceptives. *British Medical Journal (Clinical Research ed)*.

[19] Templeman C., Marshall S. F., Clarke C. A. (2009). Risk factors for surgically removed fibroids in a large cohort of teachers. *Fertility and Sterility*.

[20] Baird D. D., Travlos G., Wilson R. (2009). Uterine leiomyomata in relation to insulin-like growth factor-I, insulin, and diabetes. *Epidemiology*.

[21] Wise L. A., Palmer J. R., Stewart E. A., Rosenberg L. (2007). Polycystic ovary syndrome and risk of uterine leiomyomata. *Fertility and Sterility*.

[22] Loy C. J., Evelyn S., Lim F. K., Liu M. H., Yong E. L. (2005). Growth dynamics of human leiomyoma cells and inhibitory effects of the peroxisome proliferator-activated receptor-gamma ligand, pioglitazone. *Molecular Human Reproduction*.

[23] Clemmons D. R. (2007). Modifying IGF1 activity: an approach to treat endocrine disorders, atherosclerosis and cancer. *Nature Reviews Drug Discovery*.

[24] Campo S., Campo V., Gambadauro P. (2003). Reproductive outcome before and after laparoscopic or abdominal myomectomy for subserous or intramural myomas. *European Journal of Obstetrics & Gynecology and Reproductive Biology*.

[25] Kumakiri J., Takeuchi H., Kitade M. (2005). Pregnancy and delivery after laparoscopic myomectomy. *Journal of Minimally Invasive Gynecology*.

